# A leadership model supporting maturation of high-performance translational teams

**DOI:** 10.1017/cts.2023.598

**Published:** 2023-07-24

**Authors:** Allan R. Brasier, Shannon L. Casey, Peggy Hatfield, Patrick W. Kelly, Whitney A. Sweeney, Marin Schweizer, Bo Liu, Elizabeth S. Burnside

**Affiliations:** Institute for Clinical and Translational Research, School of Medicine and Public Health, University of Wisconsin-Madison, Madison, WI, USA

**Keywords:** Team, leadership, evaluation, competency, functional, translational team

## Abstract

Despite understanding its impact on organizational effectiveness, practical guidance on how to train translational team (TT) leaders is lacking. Previously, we developed an evolutionary learning model of TT maturation consisting of three goal-directed phases: (1). team assembly (*Formation*); (2). conducting research (*Knowledge Generation*); and (3). dissemination and implementation (*Translation*). At each phase, the team acquires group-level knowledge, skills, and attitudes (KSAs) that enhance its performance. Noting that the majority of team-emergent KSAs are promoted by leadership behaviors, we examine the SciTS literature to identify the relevant behaviors for each phase. We propose that effective team leadership evolves from a hierarchical, transformational model early in team *Formation* to a shared, functional leadership model during *Translation*. We synthesized an integrated model of TT leadership, mapping a generic “functional leadership” taxonomy to relevant leadership behaviors linked to TT performance, creating an evidence-informed Leadership and Skills Enhancement for Research (LASER) training program. Empirical studies indicate that leadership behaviors are stable across time; to enhance leadership skills, ongoing reflection, evaluation, and practice are needed. We provide a comprehensive multi-level evaluation framework for tracking the growth of TT leadership skills. This work provides a framework for assessing and training relevant leadership behaviors for high-performance TTs.

## Introduction

Team approaches are revolutionizing translational medicine because of their impact and their ability to transition across the multiple domains required for reproducible science and the sustainable implementation of health interventions into clinics and communities. Here, the application of best practices from the science of team science (SciTS) field has been used to advance the Translational Team (TT) model to address complex health interventions in order to enhance the impact of the Clinical and Translational Science Awards (CTSAs) [1–4]. We consider a TT to be a hybrid of an academic knowledge-generating team and an industry-like product development team that advances a product (device, drug, diagnostic or evidence-based intervention) into adoption by clinics or communities to improve human health [1–3]. TTs are distinct from generic interdisciplinary teams in membership composition and fluidity, simultaneous engagement in knowledge generation and product development taskwork, and operation within an academic environment.

TTs function in a complex and rapidly changing environment. Informed by real-world observations grounded in transition theory, we developed an evolutionary learning model that describes the maturation of successful TTs, focusing on three goal-directed phases – *Formation, Knowledge Generation,* and *Translation*. This model proposes that TTs develop through learning cycles, where the team acquires team-level knowledge skills and attitudes (KSAs) that enhance the major activities at each phase. These KSAs are incorporated into the team’s collective knowledge base, whose ongoing application facilitates the team’s maturation toward translation of its product into sustainable health interventions. Recognizing the phases and competencies necessary for these major performance cycles in TT development provides a framework for identifying key leadership behaviors appropriate for each phase. Specification of team-emergent processes and behaviors allows for continuous evaluation of team characteristics and performance.

This analysis draws from a large evidence base that shows how leadership plays a critical role in team performance by promoting the acquisition of team-emergent KSAs. Of the many types of leadership, functional leadership has been associated with enhanced team performance in academic, medical, and industrial contexts [5,6]. Here, we define functional leadership as the process of satisfying the team’s needs to enhance its effectiveness [5,6]. Two other leadership approaches we incorporate, transformational leadership and situational leadership, promote complementary principles. Transformational leadership, which channels team members’ intrinsic motivation by articulating organizational value and purpose, is arguably the most intensively studied leadership model linked to organizational performance [7,8]. Situational leadership involves providing tailored support by adopting different styles of leadership (e.g., directing, coaching, delegating) depending on team members’ development [9]. Note that we intentionally use the term “leadership” rather than “leaders,” because several internal sources satisfy team needs in addition to the organizing principal investigator (PI) [3].

Despite the rich evidence base that describes the dynamic impact of leadership behaviors across a range of fields from business, health care, to the military and other complex organizations, little work has focused on leadership that is applicable to TTs within the academic environment. Here, we synthesize relevant findings from the broader SciTS literature that are applicable to the major goals of each stage in the maturation of high-performance TTs. From a generic taxonomy of functional leadership, we provide an integrated framework of leadership behaviors linked to supporting the needs of TT maturation at these phases of team development. Finally, because empirical studies of TT maturation have shown that leadership styles are stable over time [3,10], we propose a multi-layered evaluation framework that reinforces continual improvement and reflection on leadership skills and practices.

## Methods

A scoping literature review was conducted in the Medline Core Collection from 2010 to 2022 according to the scoping review protocol (Supplementary File S1). From 388 citations, abstracts were selected for those that were (1). Empiric-observational; Empiric-survey, Meta-analysis, or Expert opinion/panel studies; (2). Included analysis or description of leadership; and were (3). Relevant to Knowledge-generating, product development, innovation, or translational teams. These abstracts were combined with reviews of earlier published literature [1,2]. From these, we evaluated those describing taxonomies of leadership and evidence relevant to team performance. Evaluation was performed in accordance with IRB 2017-0860-CP007 (Renewed 6/27/2022).

## Results

Earlier scoping reviews, aligned with observations of real-world CTSA TTs, have indicated that leadership behaviors impact virtually all team-emergent KSAs, and these needs evolve over time. However, empiric observations of 10 TTs within a CTSA environment have shown that leadership behaviors are stable over a 3-year observation period [3]. Therefore, a deeper understanding of the most impactful leadership behaviors is urgently needed for developing a relevant training program. Moreover, a context-sensitive and stage-relevant evaluation model will be necessary to advance the acquisition and application of leadership skills and attitudes in the TT environment. We seek to advance both of those needs.

### Leadership Behaviors and the Enhancement of Team-emergent Competencies

Earlier work by the CTSA Team Science Affinity Group identified four team-emergent competency “domains” associated with TT performance: (1). affect, (2). communication, (3). management, and (4). collaborative problem-solving [2]. Although leadership behaviors were associated with facilitating these team-based competency domains, specific behaviors that best support team competencies have not yet been fully examined. For each team-emergent competency domain, we define its foundational components and identify relevant leadership behaviors from the SciTS literature that support their acquisition.

Affect is a state where TT members share concern, empathy, and regard [11,12], which in turn increases team members’ commitment [13] and overall team performance [14]. Within this domain, we identified three foundational competencies necessary for Affect *–* (1). “trust,” the confidence that team members have in the abilities of their colleagues to do reproducible work, share results, and discuss their interpretations; (2). “cohesion,” the strength and extent of interpersonal connection between team members; and, (3). “psychological safety,*”* a shared belief that the team environment is safe for risk taking, formulating opposing ideas or challenging team assumptions [15].

Leadership behaviors promoting Affect. Establishing a culture of *trust* begins with the initial formation of the TT. Observational studies of TTs within a CTSA environment indicated that teams consist of members who have worked closely with the principal investigator (PI) in the past as well as those who have been newly recruited to the team. Consequently, for those unfamiliar with the PI, *trust* begins with building trust with leadership [16] as the first priority, which is then developed amongst other team members [17]. Studies in the SciTS field focusing on the well-studied transformational leadership model have provided evidence that practices of “individualized concern” and “respect for followers”[18] promote team *trust* [7,19], conflict resolution, and empowerment [20,21]. Initially, *trust* is exhibited by trust in the team’s leadership. The more impactful form of *trust,* inter-team member *trust* (or team trust), is established at a higher level, after trusting norms have been established and modeled by leadership behaviors [22]. Transformational leaders who understand and listen to members promote trust, conflict resolution, and empowerment [20,21]. Satisfaction with the team’s goals and perceptions of its performance were found to be associated with a multi-level model of *trust* that is exhibited as both trust in the leader as well as *trust* in the team [14,23]. In addition, positive leader-member interactions play a critical role in fostering an environment of inclusion and risk taking that is foundational for the emergence of *psychological safety*, as evidenced by a comprehensive meta-analysis of its antecedents and outcomes in ∼5,000 groups [24].

Furthering team affect, leaders who understand individual team member work styles, tendencies, strengths, and weaknesses practice inclusive leadership. Inclusive leadership promotes a feeling of team membership, which is an essential component of *cohesion* [25–27]. Similarly, transformational leaders who understand and listen to members promote trust, conflict resolution, and empowerment [20,21]. Diversity practices by the team leader have also been shown to promote affect. In a study of>4,500 health sector employees, leadership diversity practices were found to enhance *trust* and *psychological safety* in the health sector, an environment highly applicable to that of TT operations [28].

Communication is a state where the effective exchange and integration of knowledge and expertise is occurring within the TT [29]. Specific competencies within this domain include: (1). “knowledge sharing,” a behavior where team members provide shared technical information, know-how and skills relevant to advancing the team’s translational product; and (2). “transactive memory system (TMS),” a group-level understanding of “who” on the team has “what” expertise. Both *knowledge sharing* and a *TMS* interact to enhance team performance [30] and creativity [31].

Leadership behaviors promoting Communication. Team Communication is enhanced by the transformational leadership behaviors of **“**inspirational communication,” the art of expressing positive messages and statements that build motivation and confidence, and “idealized influence,” the practice of serving as a positive role model. Inspirational communication is linked with team performance and creativity, in part, by helping to establish a TMS [31,32]. Idealized influence promotes ***Communication*** by empowering team members to establish communication lines with other team members and to seek out their expertise [33]. Additional behaviors include articulating a vision and conducting team building activities that foster communication and knowledge sharing [16,34]. A study of project management teams has shown that transformational leadership behaviors promote within-team *knowledge sharing* by establishing cooperative norms for how information is exchanged [35]. Leadership behaviors that encourage members to share their knowledge are behaviors that increase the density of intra-team advice exchange networks, enabling the diffusion of adaptive, information-sharing behaviors and enhanced team performance [36].

Management is a process, largely produced by explicit leadership activities, to organize, plan and execute a TT project [37]. Specific competencies within this domain include the following: (1). establishing “roles and responsibilities;” (2). promoting “cognitive diversity” within TT membership, (3). converging on a “shared mental model (SMM*)”*; (4). “goal setting;” and (5). “project management” practices. All of these processes engender effective role identification, time management, and performance monitoring enabled by relevant and goal-aligned feedback and are associated with high team performance and satisfaction [16].

Leadership behaviors promoting “Management.” One primary objective of leadership is determining the appropriate team composition, a major determinant of effective team processes and innovation [38,39]. A SMM is foundational for enhancing team performance for the successful translation of health intervention product(s) [40,41]. Leadership behaviors have been identified as having a strong influence on the convergence of a *SMM,* a state where all team members share the same understanding of team processes, norms, and goals [40]. In particular, the transformational leadership practices of “inspirational communication” and “idealized influence” both facilitate *SMM* convergence [42–44]. In addition, leadership activities that establish norms for team interaction [45], as well as monitor member interactions, also increase team performance [46]. Leadership behaviors providing constructive and challenging feedback enable team adaptation, which also enhances long-term team performance [47–49].

Dynamic membership of TTs is one unique aspect that distinguishes them from knowledge-generating or product development teams. In TTs, scientists and trainees voluntarily engage and disengage with the core team nucleus throughout the team’s lifespan; their membership is primarily determined by the team’s current research portfolio and/or phase of translation [1,13,16]. New members can bring needed perspectives and technical talent, but their inclusion can also be disruptive to preexisting processes of team *cohesion, TMS* and *SMMs*. Leadership can reduce this disruptive impact by establishing a vetting process, such as a structured interview focusing on values, performance, and behavior, as well as by communicating team norms and expectations to all members through collaborative agreements and/or “Welcome letters” [50].

Collaborative Problem Solving (CPS) is a process that describes how the collective cognitive and social skills of the team are combined to interpret research findings, resulting in a cohesive mental representation of the problem space [51], as well as the development of novel approaches and interpretations [51,52]. Promoting *cognitive diversity* and enabling *adaptive learning* are foundational competencies for effective ***CPS***. Cognitive diversity is also strongly linked to high team performance [53–55].

Leadership behaviors promoting CPS. The transformational leadership practice of “intellectual stimulation” empowers team members to apply CPS techniques in response to disruptive events, which in turn triggers cycles of team adaptation, learning, and maturation [56]. Specifically, leadership behaviors that encourage team members to reflect, review, and/or identify deficiencies in their approach result in collective team learning [57]. Another leadership activity that supports process improvements is team briefings, which provide opportunities for collective learning and capacity development [41].

*Cognitive diversity* is advanced by inclusive leadership behaviors that foster discipline diversity in TT membership, which includes supporting the sharing of multiple viewpoints and the mitigation of hierarchal differences amongst team members [25–27]. These behaviors are essential for engaging in collective team learning, which in turn supports team goals and improves team processes [53–55]. Inclusive leadership practices are also positively linked with innovation on business teams [58,59] and potentially support stakeholder engagement in TTs [16]. One inclusive leadership approach, “perspective-taking” [60], is when leaders and the team members adopt “both-and” approaches that accept and integrate competing viewpoints, an approach shown to increase team proficiency, adaptivity, and proactivity [61]. Inclusive leadership techniques also facilitate cross-disciplinary integration [62], by finding common ground where conflicting insights can be resolved and integrated into a common *SMM*. Finally, team-focused training in knowledge sharing, critical thinking, and coordination is strongly linked to the development of CPS [51].

Adaptive learning involves team reassessments and process improvements. Sense-making is essential for how a team responds to the external disruptions (aka transition points) frequently encountered by TTs in an academic environment. Sense-making involves leadership framing a mental image of where the team is – and where they are going – in order to create an action plan, enabling productive responses to these transition points [63]. Leaders are the most important sense-makers who shape followers’ perceived meaningfulness of work-related issues [64], including their identification with a team and commitment to its goals [65]. Enhancing team members’ sense of affiliation and commitment to the team enables the team to productively respond to disruptive events [66–68], in the process turns disruption into productive activity by providing insight into the event and developing a shared path forward [69].

### Leadership Behavior Adaptation during TT Maturation

Substantial scholarship has shown how leadership styles, sources, and activities adapt to – and are influenced by – team members and processes [5]. Although transformational leadership is the most intensively studied leadership model linked to organizational performance [7,44], evidence suggests the functional leadership model is more appropriate for TTs because it embraces the concept that leadership responsibilities and behaviors vary with stages of team development [30].

Recently, we analyzed the developmental phases and temporal adaptations of TTs based on models of interdisciplinary teams aligned with real-world observations of TT maturation. This analysis resulted in a robust evolutionary learning model where TT maturation is associated with three goal-directed phases: (1). *Formation*, a phase where leadership assembles a team and develops a shared vision and plan to address a translational problem; (2). *Knowledge Generation*, a phase where the TT conducts interdependent research projects, leading to hypothesis refinement and development of a translational intervention; and (3). *Translation*, a phase where the TT engages Dissemination and Implementation Science, working with purveyors and stakeholders to sustainably implement their health intervention in the community or clinic. This model provides insight into team learning and how team-emergent competencies arise and mature. Consequently, impactful leadership behaviors that seek to promote team function must also evolve and mature. Here, we consider how these leadership behaviors align with team goals in the evolutionary team learning model, focusing on team-level leadership (as opposed to individual/dyadic interactions). In the following section, we will describe the most impactful leadership behaviors for the primary activities conducted during each stage of the evolutionary learning model of TT maturation, leading to the emergence of team-level competencies.

**Figure 1.**
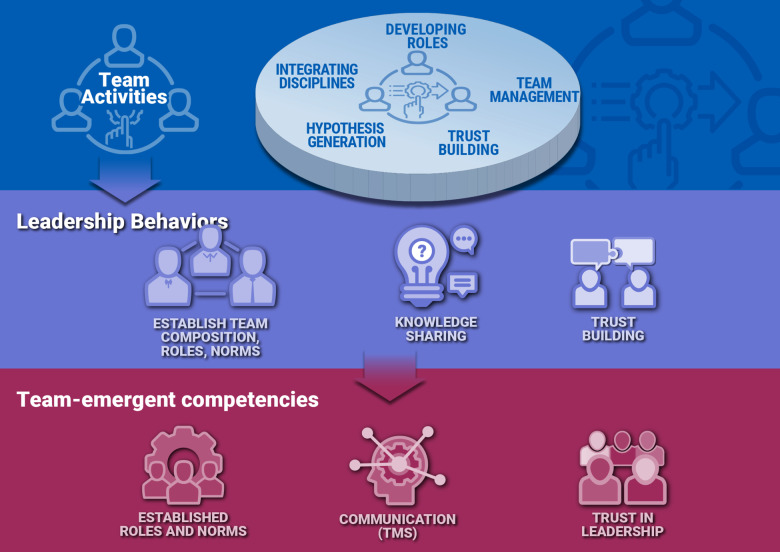
Leadership behaviors important in Translational Team (TT) *Formation*. Shown is a schematic mapping of the team activities, leadership behaviors, and team-emergent competencies during *Formation* phase. Interdependent activities primarily conducted during *Formation* are illustrated as management (recruiting members), trust building, hypothesis developing, and discipline integration. Leadership behaviors seek to satisfy the needs of the team in accomplishing the goal of *Formation.* These behaviors include team building, role definition, knowledge sharing, and establishing inter-team trust (See Table 1 for more specifics). As a result, team acquires phase-relevant competencies [knowledge, skills, and attitudes (KSAs)] linked to high performance, including affect, communication, and management [16].

Leadership behaviors during Formation. During Formation, the TT conducts a series of interrelated activities to recruit membership, integrate disciplines, define roles, establish trusting relationships driven by a charter (mission and vision), culminating in the formulation of a testable hypothesis (Fig 1). At this phase of TT development, leadership activities are initially focused on implementing effective team management practices, developing trust with team members, and establishing communication networks (Fig. 1). These leadership activities are associated with the emergence of team-level competencies in Management, Affect, and Communication (Fig. 1). Specific examples of leadership behaviors and their impact on team functioning are shown in Table 1.

**Table 1.** Leadership behaviors relevant to phases of translational team (TT) development. Key leadership behaviors are grouped by phases of TT maturation. For each behavior, the impact of this behavior on team outcome is summarized

Leadership behavior	Impact on team
Formation Phase	
Determines team composition and defines roles	Role clarity enhances satisfaction, performance, and innovation [73–75].
Formulates a shared vision	Defining shared vision is linked to team productivity [40,106,107]. Debriefs, planning, and shared mental models enhance performance [108].
Establishes expectations, goals, and timelines for taskwork	Clarifying objectives facilitates team creativity [72]. Teams with goal setting outperform those without goals [47,109]. Goal setting is energizing and directs attention [110–112]
Conducts team building activities	Promote communication (knowledge sharing, TMS, SMM) – positive work environment enabling creativity [72] Shared mental models and transactive memory are linked to enhanced team performance [48,80].
Establishes culture of inter-team trust	Understanding and listening to members promotes trust and conflict resolution [20]. Leaders resolve task-level conflict enhancing performance [55].
Manages meetings with discipline and strategy	Meeting management – agenda, punctuality – improves perceived effectiveness [70,71].
Knowledge Generation Phase	
Monitors team performance	Establishing challenging goals improves team performance [47,109]. Team process improvements (feedback, error correction) enhance outcomes [113]. Factoring individual goals and abilities is essential to collective behavior [114,115]. Involving leadership style helps to manage conflict [116].
Promotes collaborative interpretation of results	Leaders promote coordination of team processes, leading to team learning and adaptation [48,66]. Leaders facilitate adaptive learning linked to enhanced performance [117–119].
Monitors need and provide resources	Leaders stimulate helping behaviors [120]. Leaders develop team by coaching and training enhances performance [48,121]
Promotes skills acquisition/training of team members	Feedback enables adaptation and enhances long-term performance [47–49]. Developing team skills strengthens team structure and dynamics [122].
Coaches/promotes team self-management	Understanding styles, tendencies, recognizing strengths and weaknesses promote team learning and efficacy [25–27].
Manages team boundaries within larger organization	Setting boundaries enables members to identify with each other psychologically, improving team cohesiveness [123].
Translation Phase	
Expands discipline representation on team	Cognitive diversity boosts innovation, problem-solving, and collaboration within teams in complex environments [75,124–126]. Perspective-taking enhances team creativity [127].
Promotes psychological safety	Psychological safety promotes learning, risk taking, and cohesiveness [102,128]
Provides sense-making	Sense-making enables teams to respond to disruptive events [66–68]
Facilitates collaborative problem-solving	Collaboration enhances performance and collective intelligence [129]
Incorporates design for dissemination principles	Translational products are implemented and sustained in health care and community settings [81]

Leadership is critical for establishing team composition, defining roles and norms, as well as supporting day-to-day team operations. The acts of setting agendas, keeping minutes, and conducting debriefs enhance member perception of team effectiveness [70,71] as well as help to establish a positive work environment associated with team creativity [72]. Leadership activities that include setting challenging team-level goals on “how” the taskwork will be done, “who” will do it, and “when” it needs to be done are strongly associated with team performance, satisfaction, and innovation [73–75]. Specific leadership behaviors could include, but are not limited to, the co-development and adoption of team charters, operating guidelines, performance management practices, and resource allocation plans [76,77] (Table 1). These activities are encompassed in an evidence-based “Collaboration Planning” workshop [78] that also promotes team culture and the use of best practices for reproducible science, thereby addressing a currently unmet challenge of preclinical research [79].

The advancement of team Affect is promoted by team building activities establishing relationships with the team leader, as well as later, among team members. Leadership listening, as well as providing prompt feedback and being open to suggestions, promotes this culture of trust and has been shown to be critical for effective resolution of task-level conflicts and TT performance [20]. Leadership activities advancing the teams' KSAs in Communication are developed by advancing a transactive memory system (TMS). Also, leadership activities within Management, such as defining a shared vision and shared mental model (SMM), are linked to enhanced team performance [48,80]. Leaders who promote an understanding of the team’s translational project help to advance the within-team knowledge sharing network [36], which in turn promotes discipline and perspective integration [35].

**Figure 2.**
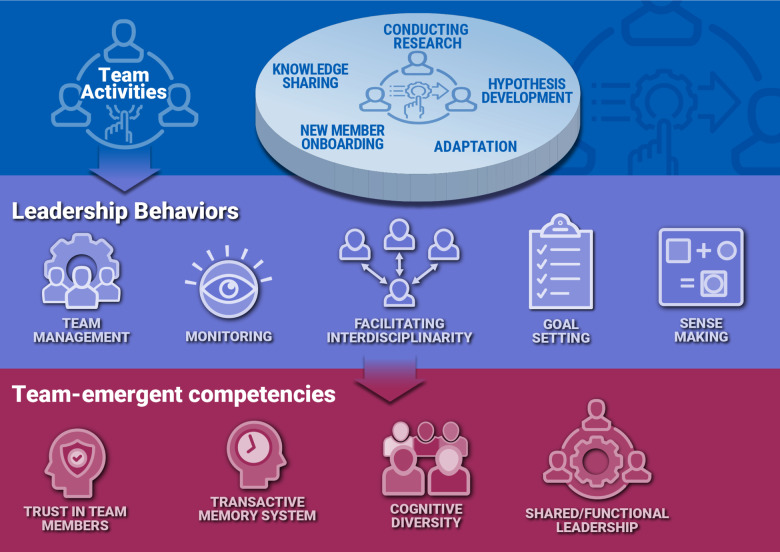
Leadership behaviors important in *Knowledge Generation*. Shown is a schematic mapping of the team activities, leadership behaviors, and team-emergent competencies during *Knowledge Generation* phase. Interdependent activities primarily conducted during *Knowledge Generation* are focused on conducting research through interdependent team member activities, developing hypotheses, adaptation, new member onboarding, and knowledge sharing. Leadership behaviors seek to satisfy the needs of the team in accomplishing the goal of *Knowledge Generation.* These behaviors include team management, monitoring, facilitating inter-disciplinarity, goal setting, and sense-making (see Table 1 for more specifics). As a result, team refines phase-relevant knowledge, skills, and attitudes (KSAs) linked to high performance, including affect, communication, and management, and acquires new KSAs in Collaborative Problem Solving and leadership.

Leadership behaviors during Knowledge Generation. During the *Knowledge Generation* phase, team members conduct activities that support research projects by developing/testing and refining hypotheses. Unanticipated experimental results can be disruptive, challenging the team to adopt new processes or experimental approaches [56], promoting new discipline involvement and sharing knowledge (Fig. 2). Leadership behaviors of team management, monitoring, facilitation of inter-disciplinarity, goal setting, and sense-making play important roles in the *Knowledge Generation* phase. These leadership activities are associated with the maturation of team-level competencies in Management, Affect, and Communication, and the emergence of Collaborative Problem and Leadership (Fig. 2; please also see Ref [56] for more detail on the evolution of team-emergent KSAs).

Leadership behaviors in team meeting management continue to be important, but as teams are conducting a research project, a key new leadership role lies in team monitoring – e.g., assessing progress toward the translational goal, tracking team motivation, procedures, and responses (Table 1). These monitoring behaviors include providing timely, specific, objective, and balanced feedback, which in turn encourages the team to review and reassess its methods, adapt to dynamic task environments, and in the process stimulating coordination and communication among team members and enhancing team performance [6]. For example, the leadership behavior of challenging members to acquire new skills to advance both individual and team goals has a positive relationship on team quality, knowledge sharing, and collective performance [39]. In addition, leadership activities that encourage and/or coach team members to get additional skills/experiences and promote sharing of this knowledge with others on the team build SMMs and expand TMSs.

Leadership practices in sense-making enable the TT to advance team-emergent KSAs in CPS. Here, leaders help teams to adapt and learn from disruptive events by developing a shared understanding, interpretation, and coping strategy for the team. Leaders who provide sense-making enhance team adaptation by communicating the presence of covert external influences and guiding the team to develop beneficial responses to them. In these activities, team members learn how their respective roles are interconnected, establishing *psychological safety*, *knowledge sharing*, and developing *TMS*s, specific team-level competencies in Affect and Communication.

**Figure 3.**
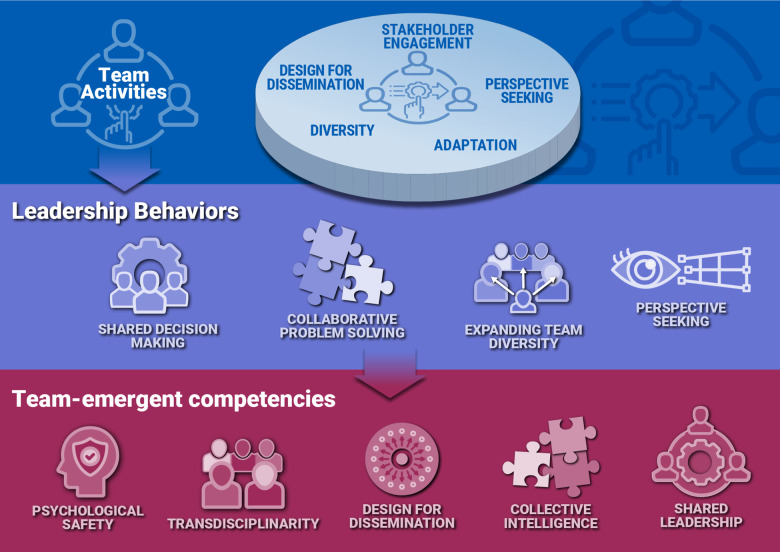
Leadership behaviors important in *Translation.* Shown is a schematic mapping of the team activities, leadership behaviors, and team-emergent competencies during *Translation* phase. Interdependent activities primarily conducted during *Translation* are illustrated as stakeholder engagement, perspective seeking, enhancing cognitive diversity, and designing for dissemination. Leadership behaviors seek to satisfy the needs of the team in accomplishing the goal of *Translation.* These behaviors include building trust/psychological safety, shared decision-making, Collaborative Problem Solving, expanding diversity, and perspective seeking. (see Table 1 for more specifics). As a result, team acquires phase-relevant knowledge, skills, and attitudes linked to high performance, including affect, communication, Collaborative Problem Solving, and leadership.

Leadership behaviors during Translation. During the *Translation* phase, teams conduct activities that include, but are not limited to, incorporating new stakeholder members and their views, establishing bi-directional communications with health systems, providers and purveyor organizations, and advancing their translational product to application in the community using Dissemination and Implementation principles (Fig. 3). In this phase, team-level leadership behaviors promote shared decision-making, collaborative problem-solving using design for dissemination [81], enhancing team diversity, and perspective seeking (Fig. 3). These activities promote the refinement of team-emergent competencies in Affect, CPS, and Leadership (Fig. 3).

**Figure 4.**
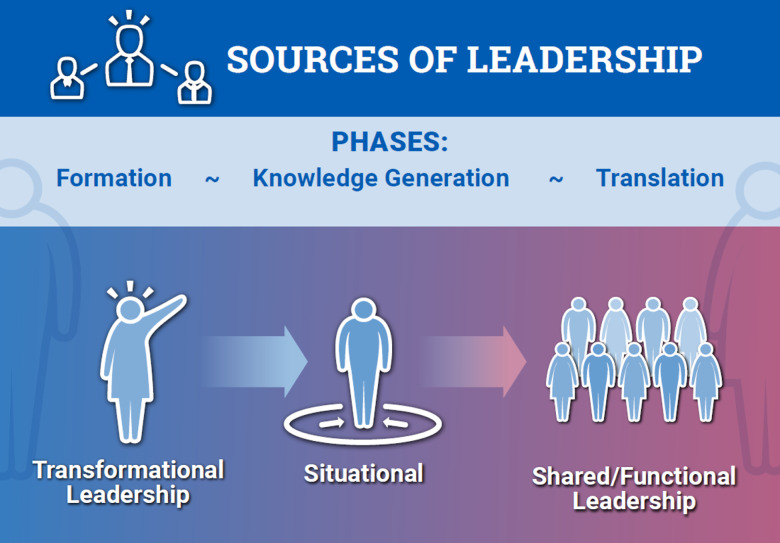
Adaptation of leadership across Translational Team (TT) maturation. Schematic map of the sources of internal leadership in a TT over the three goal-directed phases of maturation. At the initial team *Formation* phase, leadership in team building and vision development is provided primarily by the PI. As the team transitions to knowledge generation, internal members assume leadership responsibilities in domains aligned with their expertise. As team transitions to translation, the presence of psychological safety and knowledge sharing is established, and multiple sources of internal leadership can arise, leading to shared leadership.

As the team matures, the leader’s role shifts from mentor to coach and facilitator, enabling the team to transition to a shared leadership model (Fig. 4) [30]. Shared leadership is an emergent team state in which multiple members assume leadership roles, either leading one another simultaneously or by rotating leadership roles [82]. A meta-analysis found that shared leadership is linked to enhanced team function [83]. Three factors that influence the emergence of shared leadership are: (1). network leadership density; (2). decentralization of leadership, and (3). situationally aligned leadership (SAL). SAL is present when individuals with the right abilities to respond in particular situations emerge and lead the team in their areas of expertise [84]. Viewing leadership from an adaptive and dynamic model [85] of influence, Xu *et al*. [86] developed a temporal model of how leadership density, leadership decentralization, and SAL logically emerge in teams, reinforcing one another and leading to enhanced performance. These authors found that a TMS [87] serves as a key factor in the emergence of shared leadership, where the shared “meta” knowledge of team expertise sparks a team’s SAL and contributes to a more decentralized leadership network, which, in turn, increases team leadership density. The emergence of shared leadership illustrates the complex relationships between team-emergent KSAs and effective leadership.

Empiric work from SciTS has shown that shared leadership has a positive impact on team members’ innovative behavior [88] as well as their ability to overcome barriers [89]. Encouraging team self-management is associated with team member satisfaction and self-rated effectiveness [90]. Our earlier, three-year observational analysis and evaluation of TTs in the CTSA environment found that high-performance teams exhibited a co-leadership model with leadership emanating from both the PI and the early-career (KL2) trainee [3]. Functional leadership behaviors were complementary between the PI and KL2 trainee, providing a complete spectrum of leadership behaviors needed for teams through their development.

### An Integrated Model of TT Leadership

Based on this consideration of leadership behaviors supporting high-performance TTs and understanding how these change with TT maturation, we synthesized an integrated model of TT leadership by refining the applicable, generic “Functional Leadership” model. Functional leadership encompasses both “task-based” management and “person-focused” problem-solving skills, a model reinforced by an analysis of leadership behaviors across 50 empirical studies linked with team performance [19].

**Figure 5.**
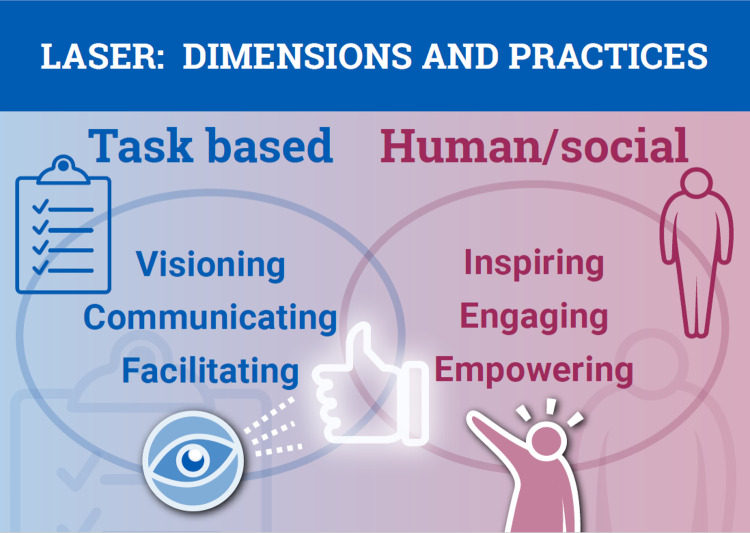
Framework for Functional Leadership across goal-driven phases. A framework for functional leadership behaviors divided into task-based and human/social-focused is used for leadership training in Leadership and Skills Enhancement for Research (LASER). This framework enables LASER to effectively teach these leadership behaviors while simultaneously mapping them to the three goal-directed phases of translational Team (TT) maturation.

Specifically, we identified task-based practices that include Visioning, Communicating, and Facilitating, whereas person-focused practices include Inspiring, Engaging, and Empowering. We define each practice below, with reference to specific observable skills that exemplify the leadership behaviors in question (Fig. 5). We accomplished this by mapping a formal taxonomy of 15 team leader behaviors, synthesized from an analysis of 517 items spanning all possible leadership dimensions, to those relevant to TTs [5]. In the following sections, we describe how this integrated model of TT leadership maps to the taxonomy of functional leadership (Table 2) and propose specific behavioral rubrics for each (Table 3).

**Table 2.** Mapping LASER practices to functional leadership taxonomy. Shown is correspondence of a generic taxonomy developed for functional leadership to LASER leadership dimensions and competency domains. Note that some dimensions of the generic taxonomy map to several LASER dimensions, providing reinforcement between the leadership practices [16]

Dimension	Generic functional leadership taxonomy	LASER
Task-Based	Compose Team	Visioning
	Define Mission	
	Establish Structure and Plan	
	Set Expectations and Goals	Communicating
	Provide Feedback	
	Manage Boundaries	
	Train and Develop Team	Facilitating
	Monitor Team	
	Conduct Taskwork	
	Solve Problems	
	Provide Resources	
Person-Focused	Apply sense-making	Inspiring
	Provide Feedback	
	Challenge Team	Engaging
	Encourage self-management	
	Promote positive social climate	
	Apply sense-making	Empowering
	Challenge Team	

LASER = Leadership And Skills Enhancement for Research.

**Table 3.** LASER leadership dimensions-practices and behaviors. Shown are leadership practices for task-based and person-based translational leadership and major TT-relevant behaviors associated with each

Dimension	Practice	Behavior
Task-based	Visioning	Works with team members and stakeholders to formulate a team vision. Analyzes the organizational context and external environmental context when formulating a team vision. Articulates a vision and its purpose to team members in a compelling manner. Monitors and ensures team membership is aligned with team’s taskwork Ensures that team members understand the team’s vision and its importance on an ongoing basis. Clarifies values, finds voice, and affirms shared values. Creates a sense of what is important, how one belongs, and what is expected for all team members. Uses core values as a guide to make critical decisions and stays focused on things that should be accomplished. Envisions the future to orient the team’s various research activities toward its long-term translational goal. Develops an ideal and unique image of the future for the common good. Helps team members to understand that each piece of the research process is an integral part of a larger vision. Gives individual team members a direction and sense as to how they can contribute to the goal.
	Communicating	Communicates actions that need to be taken by team members. Provides feedback to team members about issues related to their performance. Explains the rationale behind decisions made from team member requests. Represents the team within the larger organization. Keeps the team abreast of any changes that occur that affect them. Ensures that all members of a team possess a common understanding of the team’s approach for getting work done. Actively listens to questions and concerns from team members to help determine which projects/opportunities to pursue or pause. Creates norms for how the group will define and react to successful or unsuccessful endeavors/experiments. Requests input for the team on study design, analytic approach, and patient recruitment. Encourages team members to describe where they would like a project to go to allow team members to better take ownership of the project. Keeps the whole team apprised of various team projects.
	Facilitating	Defines each team member’s role on the project. Coaches team members to resolve issues and enhance their effectiveness. Provides resources and support to team members to enhance their skills related to taskwork on the team. Creates a culture of reproducibility, including data sharing, semi-independent replication, analysis of experimental data, and consistent documentation. Integrates new members into the team aligning them with shared vision, goals, and team norms. Provides new ideas for projects or facilitating help with any technical issues or new collaborative opportunities. Explores possibilities and invites others to freely brainstorm about how they might go about addressing a problem. Envisions the next step in the project. Debriefs successes and failures with team members. Breaks down projects into small task and celebrates the small wins.
Person_focused	Inspiring	Remains positive and sets an example as a role model. Provides team members with encouragement and emotional support for their work activities. Motivates team members to work toward achieving the vision of the team. Challenges key assumptions and status quo. Encourages self-efficacy by celebrating successes and aligning members with tasks they can successfully accomplish.
	Engaging	Fosters an open environment by involving all team members. Respects all members on the team and recognizes their contributions. Makes each team member feel like they are an important asset to the team. Encourages input from all team members how to resolve challenges facing the team. Knows the individual skill sets and strengths of various team members and what drives them to participate. Asks for feedback from stakeholders throughout the project. Ensures that all team members’ voices are heard when making decisions for the team. Expresses interest and appreciation for others' work by inviting them to talk about the work and how it contributes to the goals of the institute. Builds trust between the researchers and the community stakeholders for a project. Invites team members to participate in advanced or more challenging endeavors as a research project progresses over time.
	Empowering	Delegates responsibility for significant tasks to team members. Allows team members to make their own decisions about how to perform their work. Makes team members feel as if they have responsibility for an important part of the team’s work. Allows junior team members to take the lead on a project, from conception to execution. Encourages junior team members to pursue ideas that they initiate. Recognizes the accomplishments and contributions of individual team members by encouraging them to present scientific meetings and conferences. Provide interpretation of disruptive events to the team so that they can make sense of the situation, and adapt to the changes

LASER = Leadership And Skills Enhancement for Research, TT = translational team.

Visioning refers to leadership practices that articulate and develop a focus on the achievement of an ambitious, long-term translational goal. These practices map to the generic functional leadership taxonomy categories of “compose team,” “define mission” and establish “structure and plan” (Table 2). *Visioning* practices create a sense of what is important, how team members belong, and what is expected for all involved in contributing to the planning, development, and conduct of research. Specific behaviors describing *Visioning* practices relevant to TTs include selecting the appropriate membership for a team, aligning everyone with team goals, articulating a vision, and providing team member direction (Table 3).

Communicating are leadership practices that provide accurate, relevant, timely, and important information about tasks and actions that need to be performed by team members or changes in a project’s status that will affect the team. These map to the generic functional leadership taxonomy categories of “establish expectations and goals,” “provide feedback” and “manage boundaries” (Table 2). Communicating leadership behaviors include listening effectively to questions and concerns from team members, fostering dialogue among the team members to align their activities with the team vision, as well as providing direction for team members, giving feedback, and representing the team within the larger organizational context (Table 3).

Facilitating are leadership practices that provide direction and feedback, clarify responsibilities, and provide the necessary resources needed to support team success. These practices map to the generic taxonomy categories of “training and developing team,” “monitoring team,” managing “taskwork,” “solve problems” and “provide resources” (Table 2). Facilitating behaviors include providing the financial resources and support to team members to help them successfully complete their work as well as providing constructive feedback to team members to help them improve their performance. They also include defining team member roles on the project, integrating and aligning new members, and coaching team members to enhance their effectiveness (Table 3).

*Inspiring* involves leadership practices that create positive cognition, affect (trust/cohesion), and commitment to the team’s long-term purpose and goals. These practices map to the generic leadership taxonomy of “sense-making,” “providing feedback” and “challenging team” (Table 2). Inspiring behaviors include not only articulating examples of successes to highlight what the team values but also drawing attention to the dynamics that served to catalyze those successes, especially examples where the cooperation between multiple members led to success. They also include providing encouragement for team members, serving as a positive role model, and challenging the status quo (Table 3).

*Engaging* involves leadership practices that foster a participative and inclusive team environment and culture. These practices map to the generic taxonomy of “encouraging self-management” and maintaining a productive “social climate” (Table 2). Engaging behaviors include making each team member feel like they are an important asset to the team by, for example, asking for feedback from team members at the design and analysis stages of the project. They also include respecting input from all team members, acknowledging contributions, and fostering an inclusive environment (Table 3).

*Empowering* involves leadership practices that provide team members with the authority and autonomy to enhance their effectiveness in performing job duties. These practices map to the generic functional leadership taxonomy of “sense-making" and “challenging team” (Table 3). Empowering behaviors may allow team members to take the lead on relevant pieces of the project, encouraging them to make their own decisions about how to perform those tasks and meet the project goals. They also include providing guidance during disruptive events and building team member self-efficacy (Table 3).

### Leadership and Skill Enrichment for Translational Research (LASER) Program

Despite the evidence that leadership enhances team resilience [68], performance [91], and innovation [73–75], there are few tools available for enhancing clinical and translational research leadership. To address this gap, the Team Science program within the Institute for Clinical and Translational Research (ICTR) at UW-Madison developed and evaluated the LASER Program. Our TT phase-relevant training includes introductory concepts of TTs for early-career learners followed by training in forming, managing, participating in, and leading TTs for early-stage investigators (ESIs). For example, we outline the concepts of functional, transformational, and situational leadership. We share the 15 team leader behaviors distilled from the literature to demonstrate the rationale for shared leadership (see Table 2). Specifically, because very few leaders can consistently exhibit or embody all these varied leadership skills, shared leadership eventually becomes imperative. We also convey the important phases of TT evolution and the fact that the most crucial leadership behaviors at a given time depend on these goal-directed phases. We provide evaluation recommendations that prioritize the dynamic nature of TTs within dynamic and multi-layered environments [92]. Our ESI training focus on leadership is motivated by the National Research Council’s identification of leadership as an unmet need that has a major effect on the efficiency of team research [91].

LASER, an evidence-informed leadership training, emphasizes KSAs appropriate to each stage of the TT lifecycle while simultaneously weaving together didactic material, skill-building exercises utilizing role-playing, and problem-solving using real-world vignettes. For example, training approaches relevant to the *Formation* phase include how TTs are unique examples of teams; ideal early TT composition; building trust and psychological safety, and visioning research program mission, vision, and values. Specifically, we summarize the evidence that TTs drive innovation, improve performance (publications, patents, grants, etc.), foster reproducibility, enhance satisfaction, and impact society. We emphasize that TTs have unique challenges like diversity of members, deep knowledge integration requirements, intensive communication needs, dynamic membership, and high task interdependence. We work with ESIs to formulate their research mission and vision as well as their leadership values by hosting small breakout sessions for them to vet these concepts with peers. We also cover the important topic of setting expectations for the members of their TT, for example, agreeing on processes for sharing data, credit, and authorship.

For the *Knowledge Generation* phase of the TT lifecycle, LASER focuses on how TT leaders build capacity, set expectations, develop SMMs, and resolve conflict. We emphasize the importance of leadership tasks such as monitoring progress, managing team member boundaries, building and structuring team feedback, and challenging team members to develop new skills. We host breakout discussions prompted by real-world scenarios encouraging scholars to brainstorm what expertise might be missing from their team and how to build that capacity. We discuss the “optimal” mix of TT members including balance (newcomer/incumbent team members and junior/*s*enior researchers) and diversity that encourages early adoption of new ideas and fosters a culture of self-correction, continuous improvement, and adaptation. We help scholars identify skills/roles that may be missing from their TT particularly as they look toward the *Translational* phase of their research.

For the *Translational* phase of the TT lifecycle, we further emphasize the leadership behaviors that must be sustained and reinforced to move from research to practice. We share the theoretically grounded Leadership model for the lifecycle of TTs conveying the task-based and human/social leadership dimensions (Fig. 5) that will remain crucially important across time. We discuss important translational science skills like design for dissemination and how to access these resources locally. The LASER curriculum continues to emphasize conflict resolution techniques and methods to ensure continued trust and psychological safety as TT membership evolves to embrace shared leadership. Within LASER, ICTR’s TT leadership model guides development of context-appropriate, just-in-time training to ensure that leadership, regardless of TT phase, sustains a culture of trust, rigor, and reproducibility [78,79].

### Challenges in Leadership Training

Our earlier studies of TTs within the CTSA environment were conducted to assess dimensions of team capacity and progress across the translational spectrum, which involved a structured rubric of 4 components for each dimension assessed by reviewers external to the TT [3,4]. We noted that many of the individual components developed in sophistication over the course of the 3-year observation period. Interestingly, however, leadership skills did not change [3]. We interpreted this finding to indicate that impactful leadership training, those resulting in behavior changes, will require ongoing reflection and reassessment by leaders. Consequently, we propose using a leadership evaluation framework to assess the underlying mechanisms, within specific and variable environments (i.e., funding, institutional), that contextualize and drive leadership change.

### Leadership Evaluation

Evaluation of improvements in leadership skills based on LASER training requires attention to multiple levels of analysis, including how a trainee’s self-awareness develops, how behaviors and practices change, the impact of the growth of an ESI on the TT, and the impact on the context within which the team operates. Evaluation models for training in academic medicine frequently center Kirkpatrick (i.e., learner reactions, learner KSAs, change in learner behavior, and outcomes) or CIPP (Context, Input, Process, Product models) [93–95]. In recent years, CTSA programs are using the Translational Science Benefits Model (TSBM) [96] to better assess outcomes associated with ESI and team development. No matter which evaluation model is selected, it is critical that there is clarity in the conceptual underpinnings of LASER to properly evaluate it. Evaluators are charged with selecting from a wide array of evaluation options to capture the complex, dynamic, and temporal shifts in leadership development as an ESI’s career develops [92].

We outline here how CTSA evaluation frameworks can be operationalized with four considerations in mind: *planning*, *intervention*, *design*, and *constructs of interest* (See Table 4) [92,97]. Proactive, intentional evaluation *planning* efforts are necessary to select a priori a program evaluation model, theory of change, and focal points of the leadership curriculum [93]. In our pilot development of LASER, we planned the theoretical model, curriculum, evaluation, and theory of change. We embedded our model for training within our local strategic plan, with 13 strategic objectives (e.g., Improving evidence-based training for mentorship; developing professional development and leadership capacity), mapped to the TSBM. That process allowed for the specification of local hub resources aligned with our areas of strength, which include mentorship training, mentor-mentee alignment, health equity and disparities research training, and dissemination and implementation.

**Table 4.** Evaluation considerations across planning, intervention, design, and construct levels with operationalization for a CTSA hub

Evaluation considerations		Operationalization
Planning	Evaluation model, theory of change, curriculum, leadership competencies	Model consistent with local CTSA strategic priorities and TSBM goals; LASER objectives mapped over repeat trainings, selected leadership ability, team management ability, team practices
Intervention	Trainings, mentor conversation, commitment to change tasks	LASER dosage, mentor-mentee mapping, refined Individual Development Programs, commitment, and progress toward change
Evaluation Design	Sequential explanatory strategy (qualitative, quantitative), mixed method, longitudinal, individual + context	Annual participant interviews; bi-annual competency, and team development surveys; annual mentor-mentee leadership conversations; assessment of team member progress; end of trainee window translational team assessment of progress
Constructs of interest	Leadership KSAs and qualities, competencies, behavioral practices, team outcomes, contextual impacts	Leadership Competency KSAs over time [101]; development within specific contexts; individual and team transition, action, and interpersonal processes [130]

CTSA = Clinical and Translational Sciences Award; KSA = knowledge, skills, and attitudes; LASER = Leadership and Skills Enhancement for Research.

To evaluate ESI leader growth over time, the LASER *intervention* included didactic training, mentor conversations, trainee self-reflection, peer support, and intentional commitment to improving as a leader. Epistemological considerations were made regarding intervention dosage, mentor and peer meeting frequency, self-reflection, and commitment toward positive change, in addition to the frequency of collecting evaluation data. Because leadership change is long-term, achieved through integration of engaged behaviors and practices within relationships, we embedded leadership goals, conversation, assessment, and commitment within trainee Individual Development Plans. We recommend that leadership training programs integrate (1). occasional and ongoing meaningful conversations about leadership between trainees and trainers or mentors and (2). commitments from trainees to enact leadership skills within team settings, including revisiting progress toward that commitment. Commitment to improving as a leader was important because researchers demonstrate that conversations on anticipated behavioral change, when integrated with a commitment to change, result in both short- and long-term benefits in a variety of areas [98,99]. When a person commits to behavior with a person who presents psychological safety within the conversation, such as what happens with an ESI and members or their team, or with their own trained mentors, stronger and more sustained outcomes result. Ongoing, safe conversations and commitments to behavioral change evoke more sustained engagement of change. Conversations between trainees and mentors or program staff during the training identified the extent to which early-stage investigators were most concerned with issues of affect and communication (i.e., developing and communicating a mission/vision statement, forming a collaborative team). Providing opportunity to self-reflect over time, across the broad range of leader behaviors, is what leads to incremental and continuous change. What we learned during those conversations influenced ongoing quality improvement of the intervention.

In considering *design*, evaluation of training programs in academic medicine vary tremendously [100] in part because assessments often go beyond trainee performance data – for example to include the team or institutional context. As a result, the connections made between intervention elements and outcomes may be linear or non-linear [95] and may also be mediated by other variables. In their review of faculty development programs, Leslie and colleagues [100] found that the most popular data collection methods tend to be self-reported survey assessments of behavioral change. More rigorous evaluation methods would assess triangulating factors, including assessment of team member perspectives and organizational factors. Mixed-method designs, including interviews, allow for a nuanced understanding of program components that are most meaningful across a range of leadership styles and personalities and within diverse contexts. We elected to use mixed-method longitudinal design focused primarily on individual trainee growth, with the inclusion of mentor ratings, team perspectives, and organizational factors in the later stages of design. As leadership can be an intrinsic quality, it was important for our team to establish a baseline of pre-training capacity. As leadership is also highly context-dependent, we situated trainees within their unique context through qualitative interviews that capture the impact on teams and departments [95].

Given the complex, dynamic environment of team science, the selection of *constructs* is best considered as a reflection of the model, curriculum, and goals of the training program [100]. Attention must be paid to specific operationalization of LASER constructs. Our previous evaluations have integrated trainee-level constructs (e.g., reaction to programming, attitudes/perceptions, and knowledge/skills), trainee behaviors (self-reported or observed), and contextual or organizational outcomes such as benefits to stakeholders or practices. Scholars and mentors rated leadership competencies over time (i.e., rate your/their leadership skills: *novice, advanced beginner, competence, proficient, expert*), evidencing both variability in leader and mentor ratings and improvements in both over time. There is evidence that evaluation of psychological processes – embracing leadership skills as relevant, behaviors consistent with trainee values, and embracing change – is worthwhile. Those factors mediate the relationship between commitment to change and actual sustainable behavior. In the current context, the *constructs* we plan to evaluate are underlying mechanisms that drive leadership change [99]. For LASER, we are evaluating leader qualities, skills and attitudes, leadership values, along with behaviors and impact within the context. Retrospective assessment of team member processes will be conducted for the Transitions, Actions, and Interpersonal processes [101], as those processes best represented the leadership constructs within the LASER framework.

## Discussion

Our model is the first that specifically identifies team-level leadership behaviors and tailors them to the phases of an evolutionary learning model (including *formation*, *knowledge generation,* and *translation)*. Our model also serves as an organizing framework for the design and evaluation of training interventions that promise to enable translational scientists to effectively lead TTs. This manuscript builds on previous work from the SciTS literature on leadership taxonomies – informed by empiric observations of TTs within the CTSA context framed within a three-phase, goal-directed evolutionary learning model of TT maturation – to propose a framework for TT leadership. Here we approach the problem from a functional leadership perspective, where TT leadership is charged with identifying and satisfying a team’s needs determined by the team’s developmental phase [5]. We then highlighted relevant team-based leadership behaviors that are most directly tied to high-performance TTs framed within task-based or person-focused perspectives and linked to KSAs needed for TT maturation and performance. One important aspect of this functional team leadership model is that team leadership dynamically varies as TT needs to evolve [30]. The challenge of meeting the shifting internal and external contingencies and characteristics of TTs provides many opportunities for leadership to arise internally, and be evaluated and adapted, from within the TT membership.

We have taken the strategy of adapting a generic, evidence-based leadership taxonomy from the larger SciTS knowledge base [5] to the specific case of TTs [2]. We contend that TTs exhibit important differences from generic knowledge-generating teams or product development teams in terms of the motivations behind team member participation, simultaneous emphasis on both knowledge generation and product development, organizational (academic) environment, dynamic membership, and evolutionary maturation. Developed in detail earlier, TTs acquire team-level KSAs that enable the team to advance to the next stage of maturation by enabling the advancement of health interventions into improvement in health with an explicit focus on Dissemination and Implementation Science. Each stage in maturation is supported by specific practices that leaders can use to satisfy collective team needs. Not only is this framework useful as a guide for relevant leadership behaviors, but it can be used for leadership training as well as a guide for assessing and improving TT leadership performance.

Considerable gaps exist in our understanding of the unique interplay between teams and leadership processes [48]. Historically, within the SciTS literature, the focus of leadership analysis has primarily been on the formal hierarchy of team leadership structures or restricted to a single individual in power. This focus has occurred despite the long-recognized fact that leadership is often distributed within a team. Although multiple leadership models suggest that leadership in generic organizational teams may come from outside the team, our observations of TTs within the CTSA context indicate that the leadership is primarily internal to the team. Our model embraces multiple sources of leadership that arise internally within TTs during their maturation; from a primarily transformational and hierarchical leadership structure that is characteristic of TT “Formation” to a more diffuse, functional leadership model with internal members playing leadership roles during *Knowledge Generation* and *Translation* phases of TT maturation. This shared leadership includes that between the PI and an early-stage investigator, noted in an earlier observational analysis of high-performing teams in the CTSA context [3].

Despite extensive evaluation of the impact of transformational leadership on team performance, it most clearly impacts teams early in the *Formation* phase and during new member onboarding [10]. Thus, the transformational leadership model is of limited use for the needs of a TT over its full maturation cycle. Instead, evidence supports a functional leadership model empowering team members to meet team needs, is more relevant and focused on team performance [5,102]. As the team matures, a large evidence base indicates that shared leadership enhances performance, satisfaction, and member capacity development.

Based on our survey in SciTS literature and observations in TTs in the CTSA environment, we propose a behavioral-based model and identify rubrics associated with idealized behavior described in each. This focus on team leader behavior is advantageous because behaviors are externally observable, quantifiable, and most relevant for influencing team performance. We believe this work will enhance the opportunity for scholarship of leadership behaviors and inform strategies for training.

Although the focus of this study has been on the impact of leadership on TT performance, more work will be needed to determine the impact of leadership behaviors on other team outcomes, such as innovation (e.g., the ability to apply new technology to a different problem space), satisfaction, member capacity, and organizational effectiveness. Some of these broader impacts of leadership are illustrated in Table 1, which should be viewed as a partial listing of leadership impact. Although aspects of transformational leadership are linked to team performance, some studies suggest that this effect is indirect, by promoting a culture of knowledge sharing, and that knowledge sharing *itself* mediates the relationship between leadership and successful team performance. As an example, a study of teams driven by time-sensitive goals and requiring cooperation on interdependent tasks found that shared leadership amplifies project success directly via knowledge sharing and cohesion [103]. More studies will be required to determine whether leadership behaviors are direct or mediated by other KSAs.

We are aware of earlier work developing a training and assessment model for team leadership developed from a needs assessment of KL2 trainees [104]. However, that model is not connected to a robust assessment of team-focused competencies, nor does it account for appropriate behaviors that address distinct team goals as they mature. Nevertheless, important similarities can be found between the two approaches. For example, studies in knowledge-generating teams have suggested that the positive linkage between leadership and team innovation is mediated by the acquisition of a teamwork-based *SMM*. In addition, similarity in the mental model and team efficacy mediate the link between leadership and intra-team conflict resolution [105]. These findings do not undermine the impact of leadership in teams, but highlight the dynamic interrelationship between team activity, leadership, and outcomes embraced here. More evaluation of TTs will be required to more fully understand these interrelationships.

Advancing skills for leadership is a challenging problem. Previous observational studies of leadership behaviors within TTs in the CTSA environment over 3 years found that leadership was the team characteristic that was most resistant to change [3]. However, this observational study was limited because it did not include a focused leadership training intervention. We propose that the functional leadership approach, with emphasis on leader-team interactions that lead to enhanced team performance [5,102], will have the most impact on CTSA-like TTs. In addition to advancing a taxonomy of leadership, effective strategies for measuring and evaluating the impact of leadership training and better understanding team flow of information will be foundational to advancing evidence-based training within CTSAs. To address this problem, we propose a multi-layered evaluation plan that promotes intentional evaluation planning with triangulation and sustained commitment to behavioral change. In addition, sustained, intentional commitment to leadership skills will be necessary to have meaningful impact. Systematic application of Implementation Science in developing, testing, and disseminating team leadership training, such as the Wisconsin Interventions in Team Science approach [16], is sorely needed. We believe the model described here is a first step toward that goal.

## Conclusion

In conclusion, TT leadership dynamically evolves as the team undergoes evolutionary learning stages in its maturation. Based on research in the SCiTS field and empirical studies of TTs in the CTSA context, we propose that a task-based and human/social-focused framework for leadership behaviors relevant to the stage of TT maturation will be linked to high performance. This work will help to inform future refinement and evaluation of clinical and translational research leadership training to better address challenges faced by TTs.

## Supporting information

Brasier et al. supplementary material Brasier et al. supplementary material

## Supplementary material

The supplementary material for this article can be found at https://doi.org/10.1017/cts.2023.598.
