# Supplementary material for: A leadership model supporting maturation of high-performance translational teams
Source: J Clin Transl Sci. 2023 Jul 24;7(1):e171. doi: 10.1017/cts.2023.598 (PMC10514693; doi:10.1017/cts.2023.598)
Supplement: Brasier et al. supplementary material [file S2059866123005988sup001.docx]

**Scoping review protocol:**

(Following JBI Manual for Evidence Synthesis: Peters MDJ, Godfrey C, McInerney P, Munn Z, Tricco AC, Khalil, H. Chapter 11: Scoping Reviews (2020 version). In: Aromataris E, Munn Z (Editors). JBI Manual for Evidence Synthesis, JBI, 2020. Available from https://synthesismanual.jbi.global. https://doi.org/10.46658/JBIMES-20-12

**Title:** A leadership model for translational team performance

**Introduction:** Leadership influences many of the specific competencies needed for effective translational team performance. However the knowledge, skills and attitudes are not known.

**Definitions**

- Translational Team (TT) -an interdisciplinary team that seeks to improve human health by developing a device, intervention, drug, diagnostic .
- Competencies: knowledge, skills and attitudes demonstrated by a team.
- Team-emergent competencies: KSAs arising from team interactions.
- Performance-advancement across the translational research spectrum.
- Affect domain describing that the bonds between TT members grounded in a concern, empathy and shared regard for others.
- Communication- effectively exchanges information and integrates team member expertise to solve research problems.
- Management actions that effectively organize and sustain components of multicomponent investigation.
- Collaborative problem solving- where cognitive and social skills of the TT are used to integrate research findings and discipline-grounded interpretations into a cohesive model.
- Leadership- the process providing or supporting the cognitive, resource and affective needs for a TT.

**Details of preliminary searches-**

1. Morgeson, F.P., DeRue, D.S. & Karam, E.P. Leadership in teams: A functional approach to understanding leadership structures and processes. Journal of Management. 36, 5-39 (2010).

This work followed the principles outlined of Fleishman to develop a taxonomy for team leadership. This included a keyword search (terms included team or group, leadership, performance, and/or effectiveness) of the ISI Web of Science index to identify relevant published articles and book chapters. In addition, we manually searched the reference sections of key team leadership articles and book chapters for other publications. They reviewed the resulting 85 articles and book chapters for measures of team leadership and to identify leadership behaviors. 517 behavioral items were grouped into 15 behaviors.

**Overall review objective**- Identify what specific leadership behaviors or skills in domains most directly impact TT performance?

**Search strategy used for this review:**

A comprehensive literature review was conducted in the Medline Core Collection to update literature identified in prior reviews from 2010-2022,

Data Bases included: Current Contents Connect, Medline and Scopus Data bases.

Search strategy: ((TI=((Leadership) AND (team OR teams OR group OR groups) AND (success* OR failure* OR performance OR effective* OR innovat* OR transform* OR evaluat* OR assess* OR functional OR antecedent* OR measure* OR model OR models))) OR ((TS=((leadership) Near/7 (team OR teams OR group OR groups) Near/7 (success* OR failure* OR performance OR effective* OR innovat* OR transform* OR evaluat* OR assess* OR functional OR antecedent* OR measure* OR model OR models))) AND (SO = (JOURNAL OF CLINICAL "AND" TRANSLATIONAL SCIENCE OR JOURNAL OF MANAGEMENT OR GROUP ORGANIZATION MANAGEMENT OR GROUP ORGANIZATION STUDIES OR JOURNAL OF APPLIED PSYCHOLOGY OR LEADERSHIP QUARTERLY OR ORGANIZATIONAL DYNAMICS OR ACADEMY OF MANAGEMENT JOURNAL OR EVALUATION THE HEALTH PROFESSIONS)))) AND (PY=(2010-2023)) AND (DT=("ARTICLE" OR "REVIEW")) AND (LA=(English)) AND (SILOID=("WOS" OR "CCC" OR "MEDLINE")).

**Eligibility criteria for inclusion into the analysis:**

1. Study type: Empiric-observational; Empiric-survey, Meta-analysis, or Expert opinion/panel
2. Leadership behavior and team performance
3. Team-type relevance: Knowledge-generating, product development, innovation, translational team

**Selection and classification of articles.**  article abstracts were screened by the primary author (a list of all considered is provided in **Supplemental File #2**)

**Conflict resolution-** negotiation among authors

**How data will be presented** – Papers are grouped by primary relevance to the 5 major competency domains, and summarized in text.
